# Prokinetic effects of *Citrus reticulata* and *Citrus aurantium* extract with/without *Bupleurum chinense* using multistress-induced delayed gastric emptying models

**DOI:** 10.1080/13880209.2023.2173249

**Published:** 2023-02-02

**Authors:** Yanrong Gong, Xiaoxia Liang, Yanting Dai, Xiang Huang, Qiaozhen Su, Yan Ma, Fenglian Chen, Shuling Wang

**Affiliations:** a School of Pharmaceutical Sciences, Guangzhou University of Chinese Medicine, Guangzhou, China; b Naval Medical University, Shanghai, China; c Guangdong Provincial Hospital of Chinese Medicine, Guangzhou, China

**Keywords:** Gastrointestinal disorder, psychotherapy, traditional Chinese medicine

## Abstract

**Context:**

*Citrus aurantium* L (Rutaceae) (Au) and *Citrus reticulata* Blanco (Rutaceae) (Ci) are commonly used as couplet prokinetics and *Bupleurum chinense* DC. (Umbelliferae) (Bup) is an herbal antidepressant in traditional Chinese medicine.

**Objective:**

This study evaluates the synergistic prokinetic effects of Bup with Au and Ci in mice suffering from multistress-induced delayed gastric emptying (DGE).

**Materials and methods:**

Kunming mice were divided into four groups: control, DGE, AuCi and AuCiBup. Mice were gavaged with AuCi (14.25 g/kg) or AuCiBup (22.13 g/kg) extract for 12 days. Gastric reminder rate, intestinal driving ratio, sucrose preference and open field test were examined, and serotonin (5-HT), motilin (MTL), substance P (SP), 5-HT_4_R and c-kit were assayed. Intracellular Ca^2+^ levels in primary cultured gastric smooth muscle cells (GSMCs) were determined.

**Results:**

Both AuCi and AuCiBup treatment significantly reduced gastric residual rate (39.5% and 67.7%, *p* < 0.01). Higher serum levels of 5-HT, MTL and SP were observed in treatment groups (AuCi: 0.060 mg/L, AuCiBup: 0.089 mg/L, DGE: 0.025 mg/L, *p* < 0.01). The expression of 5-HT_4_R and c-kit in the antrum and duodenum was upregulated after treatment (AuCi and AuCiBup, 4.3-times, 2.8-times to DGE, *p* < 0.01). Medicated serums of AuCi and AuCiBup effectively increased the influx of Ca^2+^ into GSMCs *in vitro* (1.8-times, *p* < 0.01). In terms of 5-HT_4_R expression, circulatory contents of 5-HT and SP and Ca^2+^ influx, AuCiBup demonstrated better prokinetic effects than AuCi.

**Conclusions:**

These findings indicate the potential for developing combination therapy with antidepressants and prokinetics in gastrointestinal dysmotility management.

## Introduction

Food intake and food digestion, including motility of the alimentary tract, secretion and absorption, are crucial physiological events for human beings. However, an abundance of the population worldwide suffers from gastrointestinal motility disorders such as recurrent delayed gastric emptying (DGE). These disorders typically lead to debility symptoms, such as anorexia, runny stool, general malaise and loss of weight. With regard to absence of any underlying organic alteration in the upper digestive tract in most cases, these disorders are called functional dyspepsia (FD) and psychosocial factors are addressed to be involved in the pathogenesis of gastrointestinal dysmotility (Mounsey et al. 2020). A systematic review of psychotropic medications compared with placebo for the treatment of FD included three trials of tricyclic antidepressants (TCAs) and found a reduction in dyspepsia symptoms (Ford et al. 2017).

The rhythmic peristalsis of gastrointestinal muscle is controlled by specialized gastrointestinal pacemaker cells called interstitial cells of Cajal (ICC) and mediated by some neurotransmitters and hormones (Huizinga 1999). Substance P (SP) is a neuropeptide and as a modulator it plays a pivotal role in gastrointestinal functioning (El-Salhy and Spångéus 1998; Graefe and Mohiuddin 2022). Motilin (MTL) is a gastrointestinal hormone and it is cyclically released during the fasted state by Mo cells in the upper small intestine. In mammals, MTL stimulates appetite and gastrointestinal motility contributing to the movement of undigested food in these regions into the large intestine (Al-Missri and Jialal 2021). In case of disturbed gastrointestinal motility, the release of SP and MTL stimulates c-kit proto-oncogene protein (c-kit) expression in mesenchymal precursor cells and promotes their proliferation and differentiation into ICC. Serotonin (5-HT) is a neurotransmitter and a key molecule linking the nervous system with gastrointestinal function. Peripheral 5-HT activates 5-HT receptor (5-HT_4_R) in the gastrointestinal tract, thereby initiating the contractility of smooth muscles. As a consequence, ICC pacemaker activity may be generated by the influx of Ca^2+^ in smooth muscle cells due to gastrointestinal contractility (Huizinga et al. 1995).

*Citrus aurantium* L (Rutaceae) (Au) and *Citrus reticulata* Blanco (Rutaceae) (Ci) have been commonly used as couplet medicine for the treatment of gastrointestinal disorders by reinforcing digestive functions in traditional Chinese polyherbal formulations (Zheng et al. 2020; Zhu et al. 2020). *Bupleurum chinense* DC. (Umbelliferae) (Bup) is generally used as an herbal antidepressant drug (Li et al. 2021). Considering the recommendation of using TCAs before using prokinetics for FD (Mounsey et al. 2020), we examine the synergy of combination treatment with AuCi couplet and Bup on DGE mice in this article, in order to propose the potential for developing novel therapies to address the fundamental causes of gastrointestinal dysmotility.

## Materials and methods

### Plant materials and preparation of aqueous extracts

The root of Bup, the fruit of Au and the peel of Ci were provided by Zhixin (Guangzhou, China) and their voucher specimens were identified and preserved by Professor Danyan Zhang of Guangzhou University of Chinese Medicine. All these plant materials were pulverized into powder with an average particle size of 250 ± 10 μm prior to being mixed to formulate two medicinal samples, 5 g Au and 6 g Ci (AuCi), and 5 g Au, 6 g Ci plus 6 g Bup (AuCiBup). Both samples were extracted in 10 volumes of simmering water for 1 h after 30 min of maceration and the extracts were filtered and concentrated. The final concentration of AuCi was 1.91 g crude drug per millilitre and AuCiBup was 2.95 g crude drug per millilitre. *Bupleurum chinense* was also extracted as above for fingerprint analysis and named as Bup.

### Animals

Animal protocols carried out in this study were approved by the Research Ethical Committee of Guangzhou University of Chinese Medicine, and the use of mice in all experiments was in strict adherence to with the guidelines for animal use set forth by the Institutional Animal Care and Use Committee. Specific pathogen-free (SPF) female Kunming mice (body weight 20 ± 2 g) were obtained from Experimental Animal Center in Guangzhou University of Chinese Medicine (experimental animal production license number: SCXK, 2013-0002 (Guangdong); experimental animal protocol approval code: SYXK, 2018-0085 (Guangdong)) and housed in the pathogen-free animal facility and were maintained in environmentally controlled cages (temperature 24 ± 2 °C; humidity 55–65%; 12 h dark/light cycle) with free access to water and respective diet. All mice were acclimatized for one week prior to experiment, fed with a standard chow (73.5% corn, 20% wheat bran, 5% fish meal, 1% farina and 0.5% salt). During all experiments, mice were randomly divided into groups and housed alone in separate cage.

### Induction of DGE and treatment

A 30-day combined stress regimen was designed to induce DGE, in which each day mice were exposed to one unpredictable and three predictable stressors including restraint, tail shock and forced swim. In the unpredictable stress paradigm, each of the seven stressors (wet cage, body spin by lifted tail, tilted cage, tail suspension, white noise, soak in water for 3 min at 17 °C or at 42 °C) is utilized once a week, on a different day each week. For the restraint stimulus, mice were placed individually into a 50 mL Falcon tube for 1 h, and these tubes were small enough to restrain a mouse so that it is able to breathe but unable to move freely. At the start and the end of restraint, mice were subjected to tail shock for 5 min. After a 1 h break, compulsory swimming in water at 17 °C was conducted on mice. Control mice were left to move freely in their cages. The dosage of Au, Ci and Bup for mice was, respectively, converted from the maximum clinical dose for human beings (Chinese Pharmacopoeia Commission 2019). After 30-day aversive stimulation, AuCi and AuCiBup extracts were orally administered at a dose of 14.25 and 22.13 g/kg, respectively, for 12 days according to the maximum clinical dosage, meanwhile, control and DGE group mice were treated with saline. Body weight, defecation, food and water intake were recorded regularly. At the end of the experiment, mice were bleeding from abdominal aorta under anaesthesia by chloral hydrate and after perfusion with saline gastric antrum, brain and duodenum were dissected and saved at −80 °C. Liver, colon and stomach were removed from control and DGE mice and saved in 4% paraformaldehyde.

### HPLC-ELSD fingerprinting

To verify and control the quality of herbal extracts, fingerprinting analysis was conducted on AuCi, AuCiBup and Bup samples using a high-performance liquid chromatography system (Nexera X2, Shimadzu, Kyoto, Japan) equipped with autosampler, column oven, binary pump and evaporative light-scattering detector (ELSD 6000, Alltech Technology, Nicholasville, KY). Samples were purified on a D101 macroporous resin column (H 15 cm, id 15 mm). After rinsed with 200 mL water, the column was eluted with 55% and 95% alcohol subsequently and 95% alcohol eluates were harvested for chemical analysis. Samples were separated in a Phenomenex Kinetex C18 column (4.6 mm × 250 mm, 5 μm) with the mobile phase consisting of acetonitrile (A) and water (B) at a flow rate of 1.0 mL/min. The analysis was conducted with a gradient flow as follows: 0–20 min, 38–40% B; 20–30 min, 40–50% B. The injection volume was 10 μL and the temperature of column oven was 30 °C. The flow rate of nitrogen gas in the detector was 3.0 mL/min. Data were acquired and processed by LabSolutions software (Shimadzu, Kyoto, Japan).

### Sucrose preference test (SPT)

Animals were separated into individual cages for the SPT experiment on the 28th and 40th days. Two 250 mL bottles with sipper caps were marked and placed next to each other on one side of the divided wire rack, one filled with water and the other filled with 1% sucrose solution. Consumption of the two bottles was measured for 24 h and at the 12th hour positions of the bottles were switched. For analysis of relative sucrose preference, an index was calculated as: sucrose preference=*V* (sucrose solution)/(*V* (sucrose solution)+*V*(water))×100% (Meyerolbersleben et al. 2020).

### Open field test (OFT)

The OFT is performed on the 30th and 42nd days in a behavioural testing room with adjacent area outside the door for the operator. The open field arena (30 cm (*L*)×30 cm (*W*)×38 cm (*H*)) consisted of four black painted walls and a black painted floor with 25 equant squares (6 cm × 6 cm) drawn in the bottom. One hour before testing, mice were placed in the testing room for acclimatization to the room. Prior to testing each animal, the entire open-field arena was cleaned by spraying with 75% ethanol and wiping with paper towel. Each test mouse was removed from the home cage by the tail and placed onto the central zone of the open field arena. After 30 s of adaptation, the number of squares crossed (with the four paws) and the number of rears (posture sustained with hind-paws on the floor) were counted manually in duration of 3 min. All behaviour was recorded using a video camera located 40 cm above the arena.

### Histological analysis

Liver, colon and stomach were embedded in paraffin and after frozen section multiple 4 μm sections were stained with haematoxylin and eosin. Histological analysis was conducted by two experienced pathologists blinded to the study protocol under an optical microscope.

### Measurement of gastric residual volume and intestinal transit rate

The phenol red method was carried out to evaluate gastric emptying and intestinal propulsion. Mice were fasted with free access to water for 24 h after the end of dose, followed with an oral administration of 0.2% phenol red in a volume of 10 mL/kg. Twenty minutes later, mice were sacrificed and the cardia and pylori were ligated promptly prior to the dissection of stomachs and small intestines.

The gastric content was fully harvested with 10 mL of 0.1 N sodium hydroxide and centrifuged at 5000 rpm for 15 min. The supernatant (1.6 mL) was diluted in 8 mL purified water and the absorbance was measured at 560 nm measured using a UV–vis spectrophotometer. Standard samples were processed and measured likewise after the described administration procedure on normal mice following an immediate collection of the gastric content. Gastric residual rate was calculated according to the following equation:

Absorbance of test sample/absorbance of standard sample× 100%.

The small intestine was stretched on the bench and its total length was measured from the pyloric sphincter to the caecum. A small opening was cut on the small intestine and drops of 0.1 N sodium hydroxide were added from the opening to turn phenol red into purple. The distance travelled by phenol red was measured and intestinal transit rate was expressed as percentage of the propulsion distance to the total length of small intestine.

### Enzyme-linked immunosorbent assay (ELISA)

ELISA was performed to determine MTL and SP concentrations in serum with ELISA kits (DonggeBoye, Beijing, China) and also used to evaluate 5-HT levels in serum and brain homogenate (Cusabio, Wuhan, China). Absorbance was measured on a microplate reader (Multiscan GO, Thermo Scientific, Yokohama, Japan). 5-HT, MTL and SP levels were calculated using standard curves method.

### Western blot analysis

Mouse gastric antrum and duodenal tissues (about 50 mg) were homogenized and lysed in 1 mL ice-cold RIPA buffer containing 2 mM PMSF (Beyotime, Shanghai, China). After centrifugation at 12,000 rpm for 10 min at 4 °C, the supernatant was collected and total protein concentration was determined by BCA Protein Assay Kit (CoWin Biosciences, Beijing, China). The protein was denatured by boiling for 5 min prior to undergoing 10% polyacrylamide gel electrophoresis, and was then transferred onto PVDF membranes (Bio-Rad, Hercules, CA). After blocking in 5% nonfat dry milk solution (Bio-Rad, Hercules, CA) for 2 h, the membranes were probed with primary antibodies against GAPDH (1:5000, Proteintech, Wuhan, China), C-kit (1:1000, CST, Boston, MA), 5-HT_4_R (1:1000, Proteintech, Wuhan, China) overnight at 4 °C. Membranes were washed and incubated with HRP-conjugated anti-rabbit antibody (1:3000, CST, Boston, MA) for 2 h at room temperature. The protein was visualized using an Immun-Star WesternC Chemiluminescence Kit (Bio-Rad, Hercules, CA). The protein expression was observed using Molecular Imager VersaDoc MP 4000 System (Bio-Rad, Hercules, CA) and quantified by normalizing to GAPDH. Two-colour pre-dyed proteins (10–260 kDa, Bio-Rad, Hercules, CA) were used as molecular weight markers.

### Quantitative real-time PCR

Total RNA was extracted from gastric antrum and duodenal tissues using TRIzol reagent (Takara, Kyoto, Japan) according to the manufacturer’s instructions. The total RNA was reverse-transcribed using a PrimeScript RT reagent Kit with gDNA Eraser (Takara, Kyoto, Japan) following the manufacturer’s protocol. The resulting cDNA was amplified using a 7500 real time PCR system (Applied Biosystems, Bedford, MA) with SYBR Premix Ex Taq (Takara, Kyoto, Japan). The following primers were designed and synthesized by BGI (Shenzhen, China): 5-HT_4_R forward primer 5′-AGGTCCGTGGAGAAGGT CGTG-3′, 5-HT_4_R reverse primer 5′-CACAGCCACCATCACCAGCAG-3′; c-kit forward primer 5′-CAACGGCACGGTGGAGTGTAAG-3′; c-kit reverse primer 5′-AATGAGCAGC GGCGTGAACAG-3′; GAPDH forward primer 5′-A TTCAACGGCACAGTCAAGG-3′ GAPDH reverse primer 5′-CGCTCCTGGAAGATGGTGAT-3′ GAPDH was used as an internal standard and the relative level of gene expression was calculated with 2^–ΔΔCT^.

### Primary culture of gastric smooth muscle cells (GSMCs)

Neonatal Sprague-Dawley (SD) rats (1–3 days old) were provided by Experimental Animal Center in Guangzhou University of Chinese Medicine. Animals were euthanized by inhalation of CO_2_ for at least 5 min. After confirmation of euthanasia, the stomach was immediately excised and put into pre-cold Hank’s balanced salt solution (Hyclone, Logan, UT) containing 1% penicillin and 1% streptomycin. Along the longitudinal axis of the greater curvature, the stomach was opened and washed with Hank’s buffer. The mucosa and serosa were carefully removed under Stereo Microscope (XTZ, Shanghai, China), and the muscle strips were collected and cut into 1–3 mm^2^ pieces prior to incubation at 37 °C for 1 h in digestion solution containing 1% collagenase (type I, Sigma, St. Louis, MO). Thereafter, the digestion solution was filtered through 200-mesh cell sieve and centrifuged at 1000 rpm for 5 min. The pellet was washed with Dulbecco’s modified Eagle medium (DMEM, Gibco, Carlsbad, CA) and centrifuged to eliminate broken cells and organelles. Cells were resuspended in DEME with 10% foetal bovine serum (FBS) and grown at 3.2 × 10^3^ cells/cm^2^ at 37 °C in humidified 5% CO_2_ and 95% air (Al-Shboul et al. 2018; Saponara et al. 2019; Zhang et al. 2019). One hour later, the cell suspension was transferred into a new flask and purified GSMCs were harvested owing to differential cell adhesion. Cells grown to 80% confluent were passaged using the standard trypsin/EDTA treatment. Second-generation cells were viewed using a ×20 objective of an Eclipse TS100 inverted microscope (Nikon, Tokyo, Japan), and cell images were acquired using a Canon digital camera (Canon, Tokyo, Japan).

### Immunological fluorescence assay (IFA) for authentication of GSMCs

2.5 × 10^4^ GSMCs in logarithmic phase were planted on a 24-well plate with a chamber slide in each well. Cells were cultured for 12 h, washed with PBS three times and fixed in 4% paraformaldehyde (Leagene, Beijing, China) for 20 min. After washed with PBS (Hyclone, Logan, UT), cells were incubated with 0.5% Triton X-100 (Solarbio, Beijing, China) for 10 min, washed with PBS again, and then blocked with goat serum (Boster, Wuhan, China) for 30 min. Cells were incubated with rabbit anti-mouse smooth muscle actin specific antibody (alpha-SMA, Proteintech, Wuhan, China, lot: 00056560) at dilution of 1:200 at 4 °C overnight. After washed with 0.1% PBST three times, cells were incubated with Alexa Fluor 488-conjugated Affinipure Goat Anti-Rabbit IgG (H + L) (Proteintech, Wuhan, China, lot: 20000098) at dilution of 1:200 in the dark for 1 h, and then stained with 4′,6-diamidino-2-phenylindole (DAPI, Beyotime, Shanghai, China). Each chamber slide cell side was placed down on a 100 μL drop of Fluoromount-G™ mounting medium (Yeasen, Shanghai, China) on a coverslip and mounted with nail polish. Cells were observed under a BDS200-FL inverted fluorescence microscope (Optec, Chongqing, China) and recorded with ZOE Fluorescent Cell Imager (Bio-Rad, Hercules, CA).

### Preparation of medicated serum

Twenty-four SD rats (200 ± 5 g) were randomly divided into vehicle-treated, AuCi-treated, AuCiBup-treated and domperidone-treated groups. AuCi and AuCiBup extracts were administered at a daily dose of 11.03 and 17.33 g/kg, respectively, domperidone was dosed 10 mg/kg twice per day, and vehicle-treated rats received distilled water. All administration was in duration of three days. One hour after the last administration, the blood was sampled from abdominal aorta under anaesthesia by chloral hydrate, centrifuged at 3000 rpm for 20 min, and the serum was collected, filtered through 0.22 μm millipore filter membrane and stored at −80 °C. Prior to administration to cells, both AuCi and AuCiBup medicated serums were diluted into concentrations of 5%, 10% and 20% (v/v) with DEME (Gibco, Carlsbad, CA) containing 10% FBS. Vehicle and domperidone medicated serums were diluted into a concentration of 10% (v/v) with the same medium.

### Cell counting kit-8 (CCK-8) assay

Cell proliferation and viability were evaluated by CCK-8 assay (Dojindo, Minato, Japan). GSMCs were seeded on 96-well plate (100 μL, 3 × 10^3^ cells/well), allowed to be adherent, and then treated with 300 μL diluted medicated serum, including 10% vehicle-treatment, 10% domperidone, 5%, 10%, 20% AuCi or AuCiBup. The experiment was performed in hexaplicate. After incubation with medicated serum for 12 h, 24 h or 36 h, 10 μL CCK-8 reagent was added to each cell and allowed to react for 1 h. The OD value at 450 nm was measured by an AMR-100 microplate reader (Allsheng, Hangzhou, China).

### Intracellular Ca^2+^ measurement using confocal imaging

Cells at logarithmic growth stage were seeded on confocal dishes and cultured for 12 h. After washed three times with D-Hank’s solution, cells were incubated with 500 μL Fluo-3 AM (Beyotime, Shanghai, China) for 40 min in the dark at room temperature. Confocal dishes were fixed on the stage of a laser scanning confocal microscope (LSM800 with airyscan, Zeiss, Oberkochen, Germany) and the fluorescent intensity baseline (FI_0_) of cells was measured. Cells were exposed to 100 μL 10% of diluted medicated serum and the maximum fluorescent intensity (FI_max_) was recorded during continuous image scanning. ΔFI was calculated with the formula ΔFI = FI_max_ – FI_0_ to evaluate intracellular Ca^2+^ levels. Samples were analysed in pentaplicate.

### Statistical analysis

Statistical analyses were performed by one-way ANOVA, followed by the *post hoc* Tukey’s test, or by the unpaired *t*-test unless otherwise indicated. All data are expressed as the mean ± standard deviation (SD) and plotted using GraphPad Prism 7.0 (GraphPad Software, San Diego, CA). *p* Values of less than 0.05 were considered statistically significant.

## Results

### Characterization of main compounds in AuCi, AuCiBup and Bup

**Figure 1.**
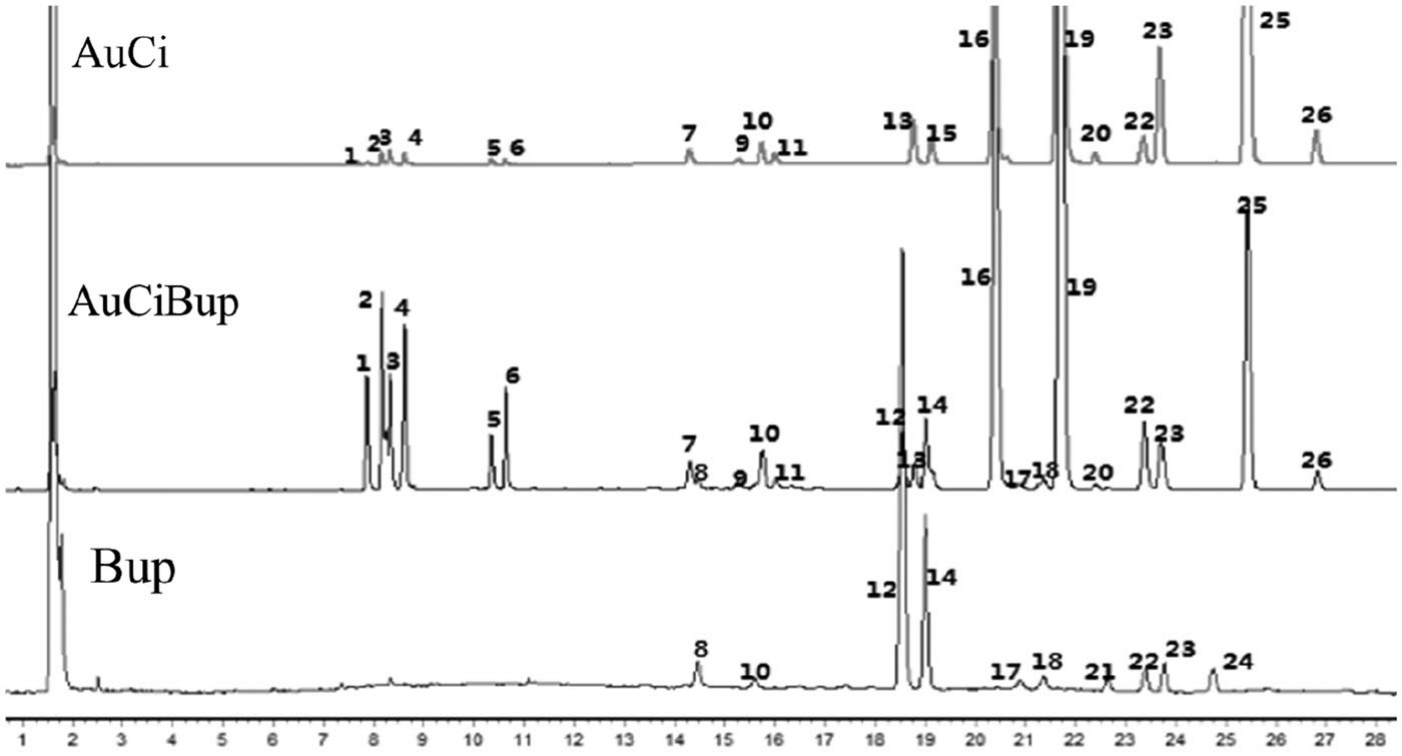
Extracts of AuCi, AuCiBup and Bup were subjected to chemical composition analyses by high-performance liquid chromatography with an evaporative light scattering detector. Peak 2: naringin, *t*_R_=8.3 min; peak 3: hesperidin, *t*_R_=8.4 min; peak 4: neohesperidin, *t*_R_=8.5 min; peak 12: saikosaponin a, *t*_R_=18.7 min; peak 14: saikosaponin b2, *t*_R_=19.2 min. Au: *Citrus aurantium*; Ci: *Citrus reticulata*; Bup: *Bupleurum chinense.*

A simple and reliable HPLC-ELSD method was developed and validated for standardization and quality control of herbal extracts through establishing chromatographic fingerprint. As shown in Figure 1, in the HPLC fingerprint, 26 peaks with relatively high intensity and satisfactory resolution were selected to represent characteristic chemical composition of samples of AuCi, AuCiBup and Bup. Five of them were identified by comparing their retention times with those of standard compounds, i.e., naringin (peak 2), hesperidin (peak 3), neohesperidin (peak 4), saikosaponin a (peak 12) and saikosaponin b2 (peak 14). Comparative fingerprint profiling indicated main compounds in AuCi and Bup samples were detectable in the AuCiBup sample.

### Stress-induced exacerbation of delayed gastric emptying was transferable via AuCi or AuCiBup treatment

**Figure 2.**
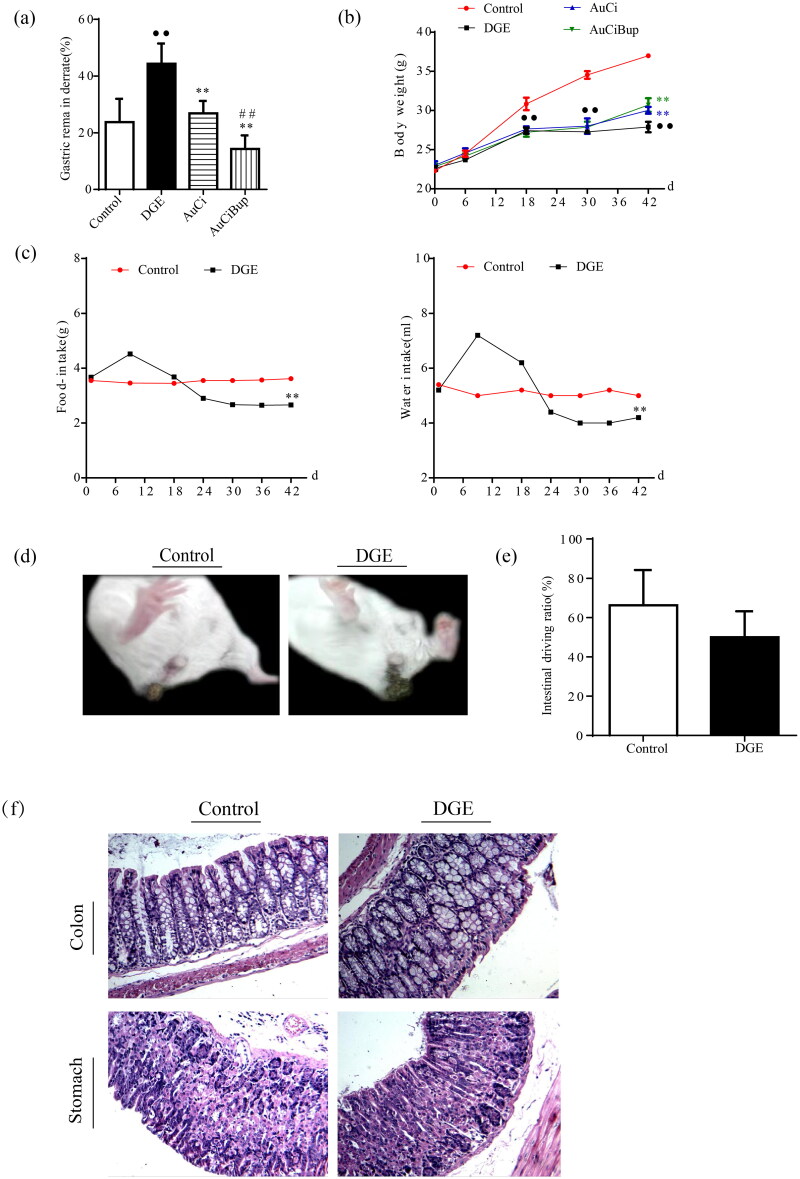
The comparison of body weight and gastric remainder rate among the four groups of mice: normal mice (control), aversive stimuli induced delayed gastric emptying (DGE), DGE + AuCi treatment (AuCi), DGE + AuCiBup treatment (AuCiBup). (a) Gastric remainder rate (%) and (b) body weight gain. (c) Aversive stimuli caused a decreased consumption of food (left) and water (right) in DGE mice. (d) DGE mice (right) dropped loose faeces. (e) Intestinal transit rate (%) was determined by the phenol red method. There was no significant difference between control and DGE mice. (f) Histological images (×100) of the colon and the stomach. Gastric emptying was measured 24 h after the last administration of each drug using the phenol red method. Values are mean ± SD. ^••^*p*< 0.01 vs. control mice; **p< 0.01 vs. DGE group; ^##^*p*< 0.01 vs. AuCi group. *N* = 6 per group.

Gastric emptying was severely delayed in mice subject to combined stresses paradigm (Figure 2(a)). Besides, the intensive stimuli caused dramatic body weight loss (Figure 2(b)), decreased food intake and water consumption (Figure 2(c)) and even led to lose faeces (Figure 2(d)) in DGE mice. Nonetheless, unimpaired intestinal transit function was observed (Figure 2(e)) and no organic lesion was found in the colon and stomach of by histochemical analysis (Figure 2(f)) in DGE mice. Either AuCi or AuCiBup treatment significantly improved weight gain and reduced gastric residual rate. Especially mice exposed to AuCiBup demonstrated a remarkable gastric residual decrease compared with mice exposed to AuCi (Figure 2(a,b)), which was suggestive of a consolidated potency of AuCiBup in promoting gastric motility.

### AuCi and AuCiBup improved psychosocial states of DGE mice

**Figure 3.**
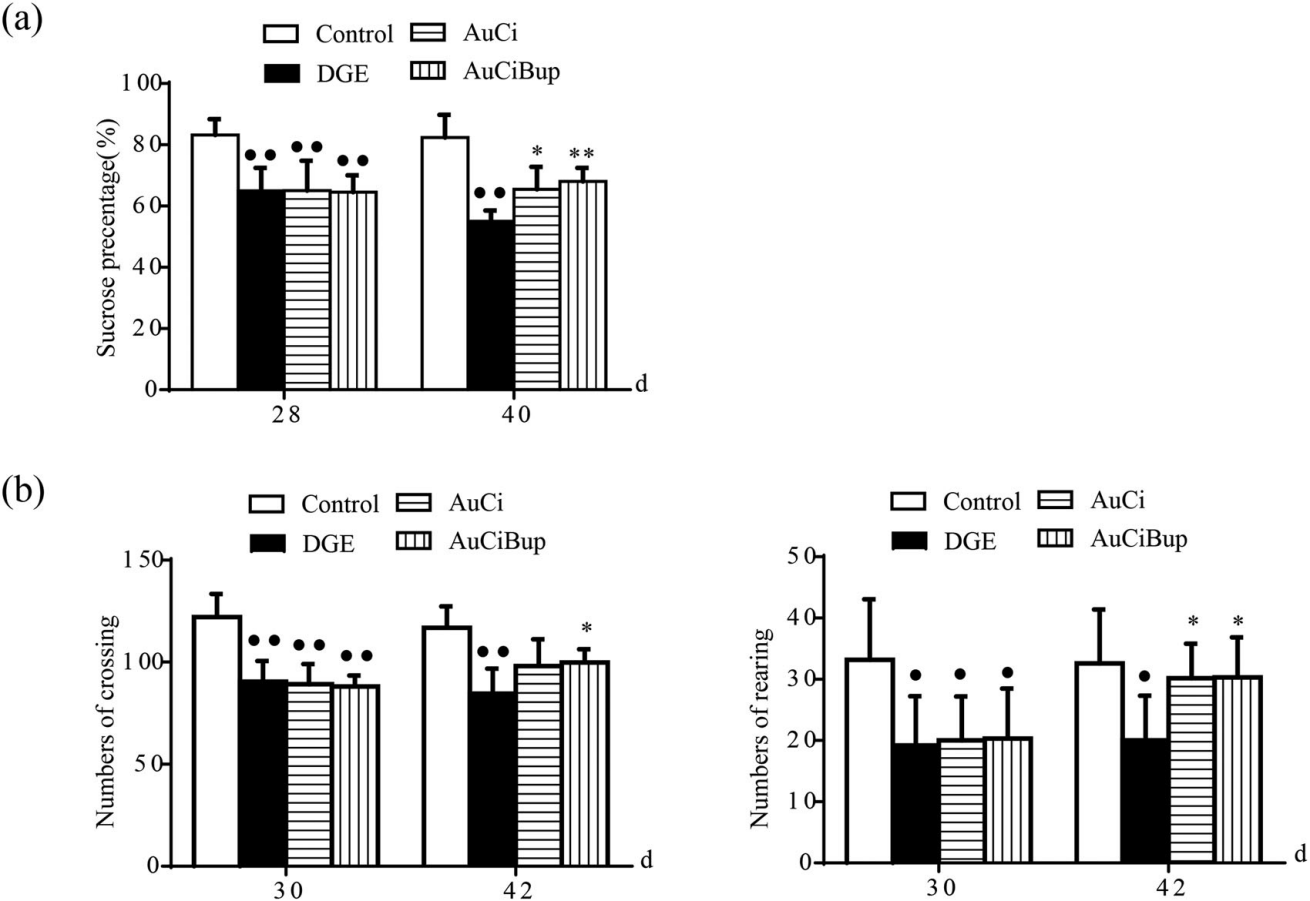
Effects of AuCi and AuCiBup treatment on DGE mice in the sucrose preference test (a) and the open field test (b). Sucrose preference index (%) was calculated to estimate the relative preference of test animals for sucrose to water. The open field test was conducted in a 30 × 30 × 38 box to analyse locomotion and stereotypical behaviours in mice with a camera used to monitor movement from the central square into other squares (b, left) as well as rearing (b, right) in test animals. Values are mean ± SD. ^•^*p*< 0.05, ^••^*p*< 0.01 vs. control mice; *p< 0.05, **p< 0.01 vs. DGE group. *N* = 6 per group.

Two behavioural assays, SPT and OFT, were carried out to study the neurobiological basis of DGE. The SPT was used to measure the relative preference for sucrose and the OFT was used for assessment of locomotive activities in mice. Changes in sucrose preference and locomotion could be indicative of altered neurological processes and might also be used to assess general health and well-being of the test animal. Owing to being stressed, DGE mice showed decreased sucrose preference and less activity in the open field compared to control mice (Figure 3, *p*< 0.01, *p*< 0.05). Either AuCi or AuCiBup treatment remarkably increased consumption of sucrose solution (Figure 3(a), *p*< 0.05, *p*< 0.01) and rearing numbers (Figure 3(b), right, *p*< 0.05). Mice exposed to AuCiBup tended to move more within the test area than AuCi-treated mice (Figure 3(b), left, *p*< 0.05).

### AuCi and AuCiBup elevated 5-HT levels and 5-HT_4_R expression

**Figure 4.**
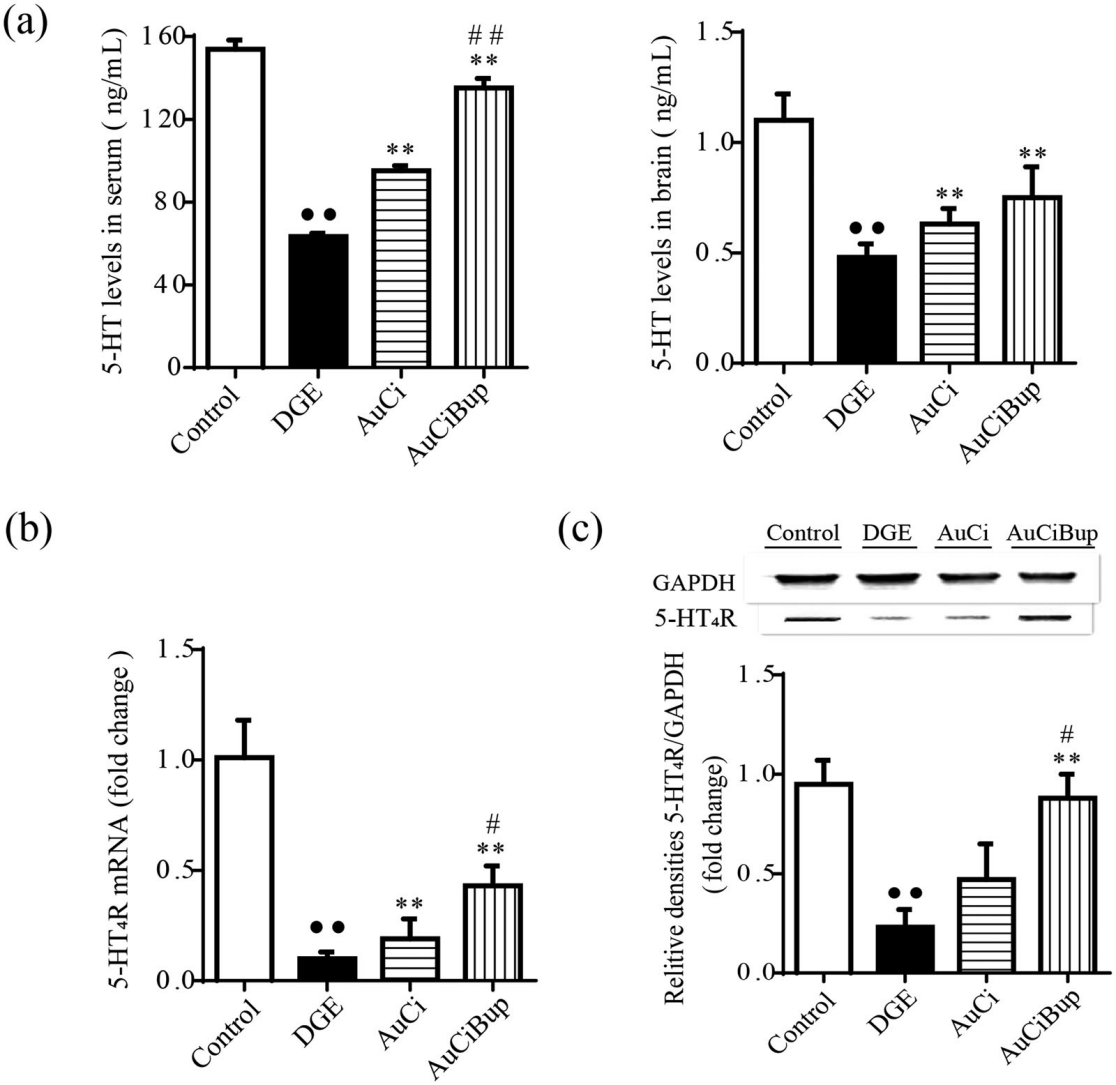
Effects of AuCi and AuCiBup on 5-HT levels and 5-HT4R expression in mice. 5-HT concentrations in serum (a, left) and brain (a, right) was determined with ELISA. The relative mRNA expression of 5-HT4R in the antrum and duodenum was assayed with RT-PCR (b) and western blot (c). GAPDH was used as the internal reference gene. Values are mean ± SD. ^••^*p*< 0.01 vs. control mice; **p< 0.01 vs. DGE group; ^#^*p*< 0.05, ^##^*p*< 0.01 vs. AuCi-treated group.

As a critical neurotransmitter in brain–gut axis, 5-HT plays a variety of regulatory effects in regulating gastrointestinal motility and sensibility by binding to its specific receptor 5-HT_4_R. As shown in Figure 4, the combined stress regimen induced decreased 5-HT concentrations in the brain and serum and 5-HT_4_R expression in the antrum and duodenum of mice (*p*< 0.01 vs. control mice). Both AuCi and AuCiBup significantly elevated 5-HT levels in the brain and serum and upregulated the mRNA expression of 5-HT_4_R in the antrum and duodenum (*p*< 0.01 vs. DGE group). Additionally, AuCiBup significantly improved the protein expression of 5-HT_4_R in the antrum and duodenum (*p*< 0.01 vs. DGE group). In this case, AuCiBup treatment demonstrated advantaged effects on the level of serum 5-HT (*p*< 0.01) and 5-HT_4_R expression (*p*< 0.05) over AuCi treatment.

### AuCi and AuCiBup enhanced the levels of substance P and motilin in the serum

**Figure 5.**
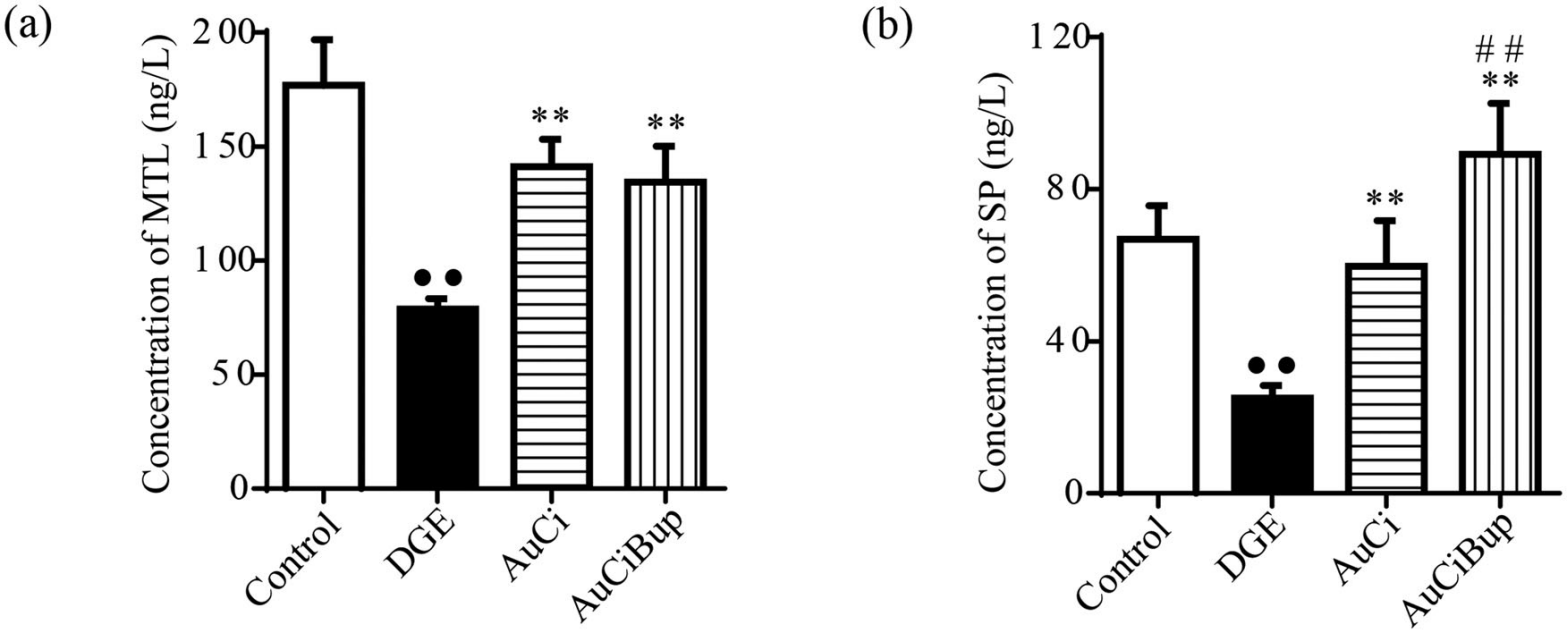
Effects of AuCi and AuCiBup on contents of MTL and SP in serum. (a) The comparison in the level of serum motilin (MTL, ng/L). (b) The comparison in the level of substance P in the serum (SP, ng/L). The contents of MTL and SP were analysed via ELISA. Values are mean ± SD. ^••^*p*< 0.01 vs. control mice; **p< 0.01 vs. DGE group; ^##^*p*< 0.01 vs. AuCi-treated group.

Motilin is a gastrointestinal hormone, SP is a neurotransmitter, and both of them play important roles in regulating gastrointestinal motility. As shown in Figure 5, the aversive stimuli exerted on mice dramatically reduced the concentrations of serum MTL and SP, whereas AuCi and AuCiBup remarkably elevated their contents. Additionally, AuCiBup exerted a much greater impact on serum level of SP than AuCi (*p*< 0.01) presumably due to the pharmaceutical effect of Bup on nervous system.

### AuCi and AuCiBup upregulated c-kit expression in gastrointestinal tissue

**Figure 6.**
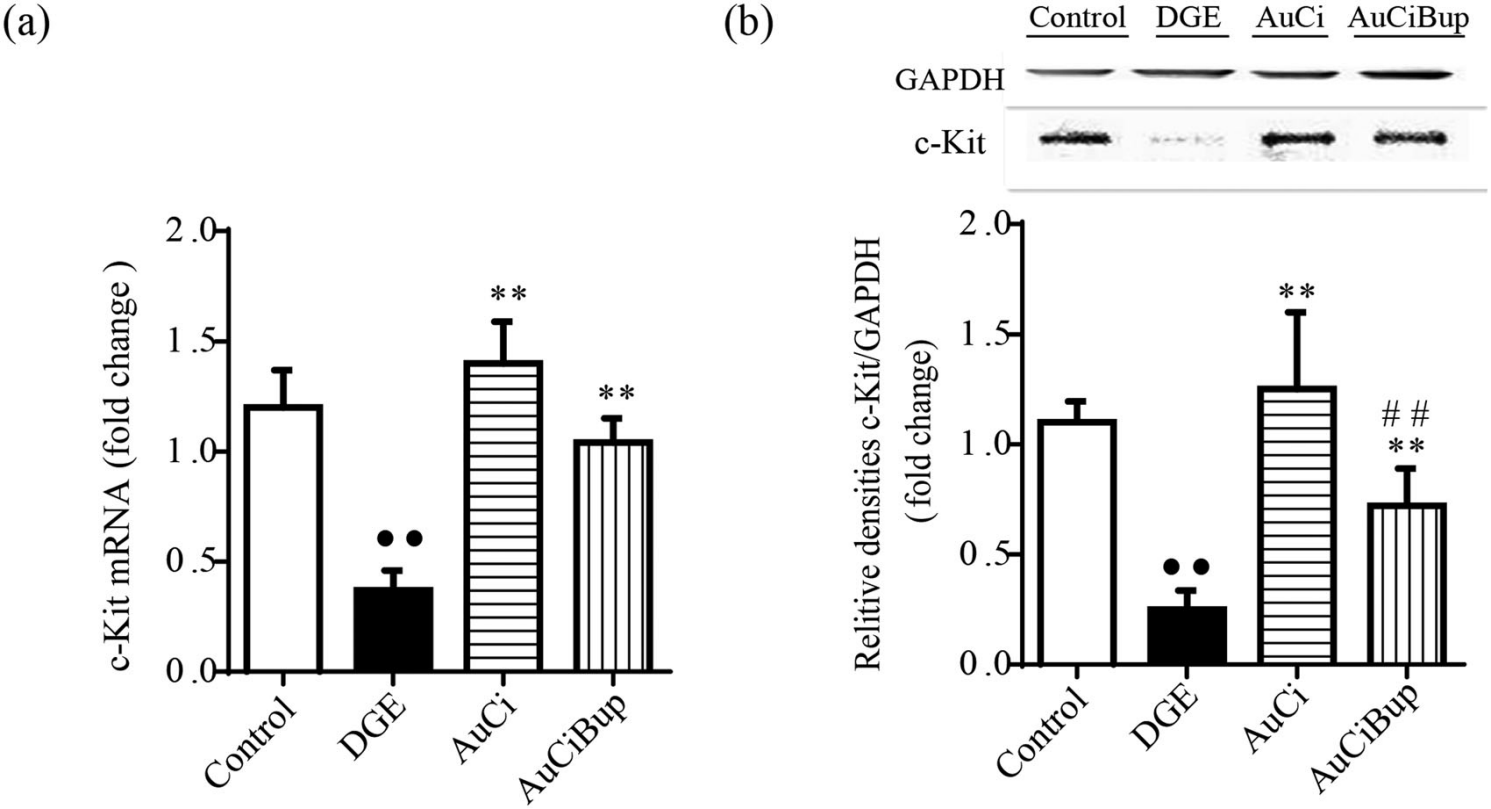
Effects of AuCi and AuCiBup on c-kit expression in mice. The relative mRNA expression of c-kit in the antrum and duodenum was assayed with RT-PCR (a) and western blot (b). GAPDH was used as the internal reference gene. Values are mean ± SD. ^••^*p*< 0.01 vs. control mice; **p< 0.01 vs. DGE group; ^##^*p*< 0.01 vs. AuCi group.

As a specialized gastrointestinal pacemaker, ICC represents a potentially valuable therapeutic target of gastrointestinal dysmotility. c-kit expressed on the membrane of ICC is considered to be their critical marker. The combined stresses paradigm notably reduced the gene and protein expression of c-kit in gastrointestinal tract (approximately cut down to one third), whereas either AuCi or AuCiBup treatment tempered this alteration and even made a reversal (Figure 6). This provided evidence that AuCi and AuCiBup facilitated an efficient movement of contents through the gastrointestinal tract.

### Medicated serums of AuCi and AuCiBup increased the influx of calcium ions into GSMCs

**Figure 7.**
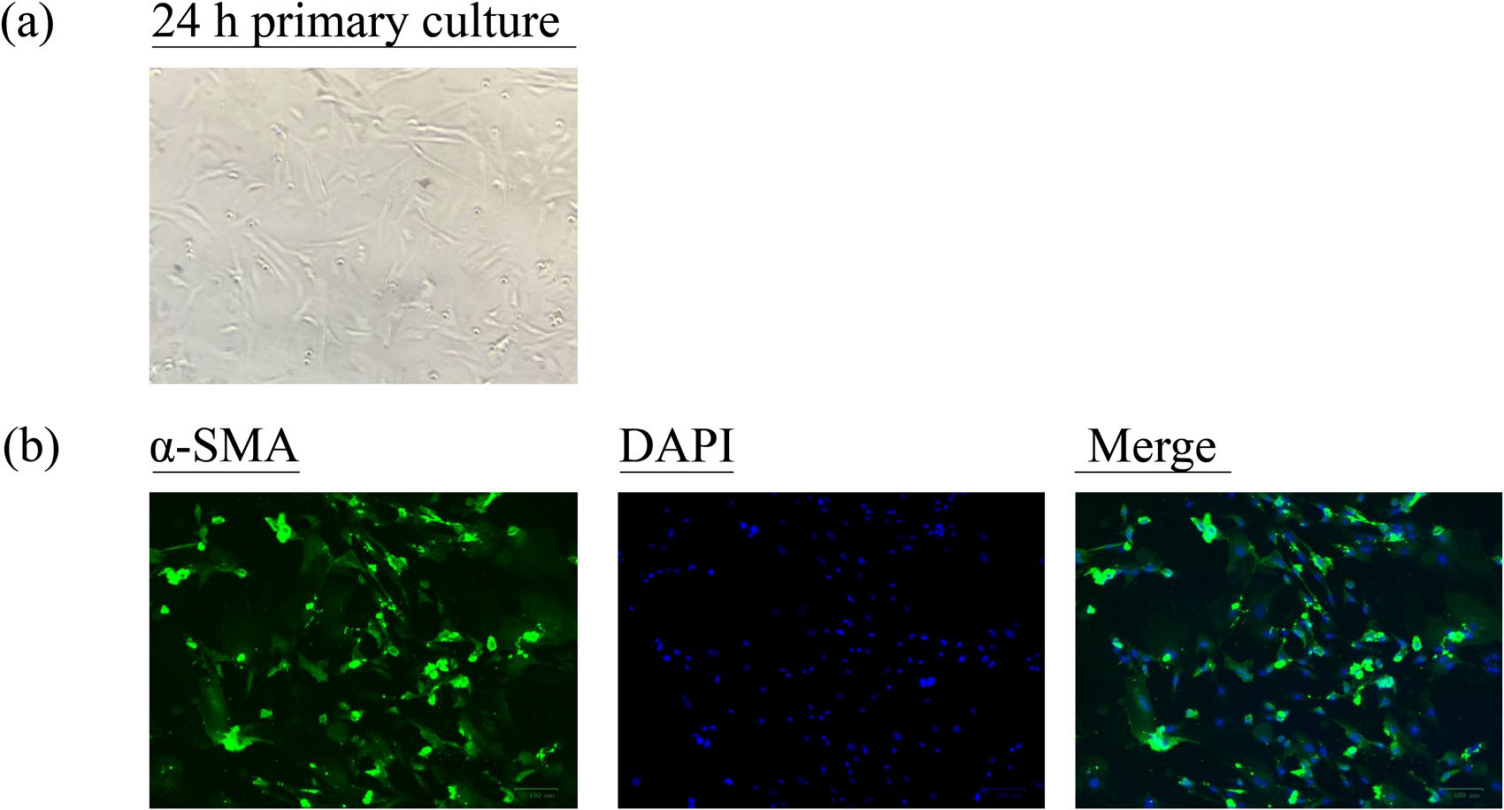
Characterization of rat primary GSMCs. (a) The isolated GSMCs were totally adherent and showed spindle-shaped, fibroblast-like morphology after 24 h of primary culture. (b) The cells underwent a specific α-SMA staining with Alexa Fluor 488-conjugated affinipure following with a nonspecific DAPI staining. An inverted fluorescence microscope was used to observe the cells. Scale bar = 100 μm (original magnification ×200). DAPI: 4′,6-diamidino-2-phenylindole; α-SMA: α-smooth muscle actin.

**Figure 8.**
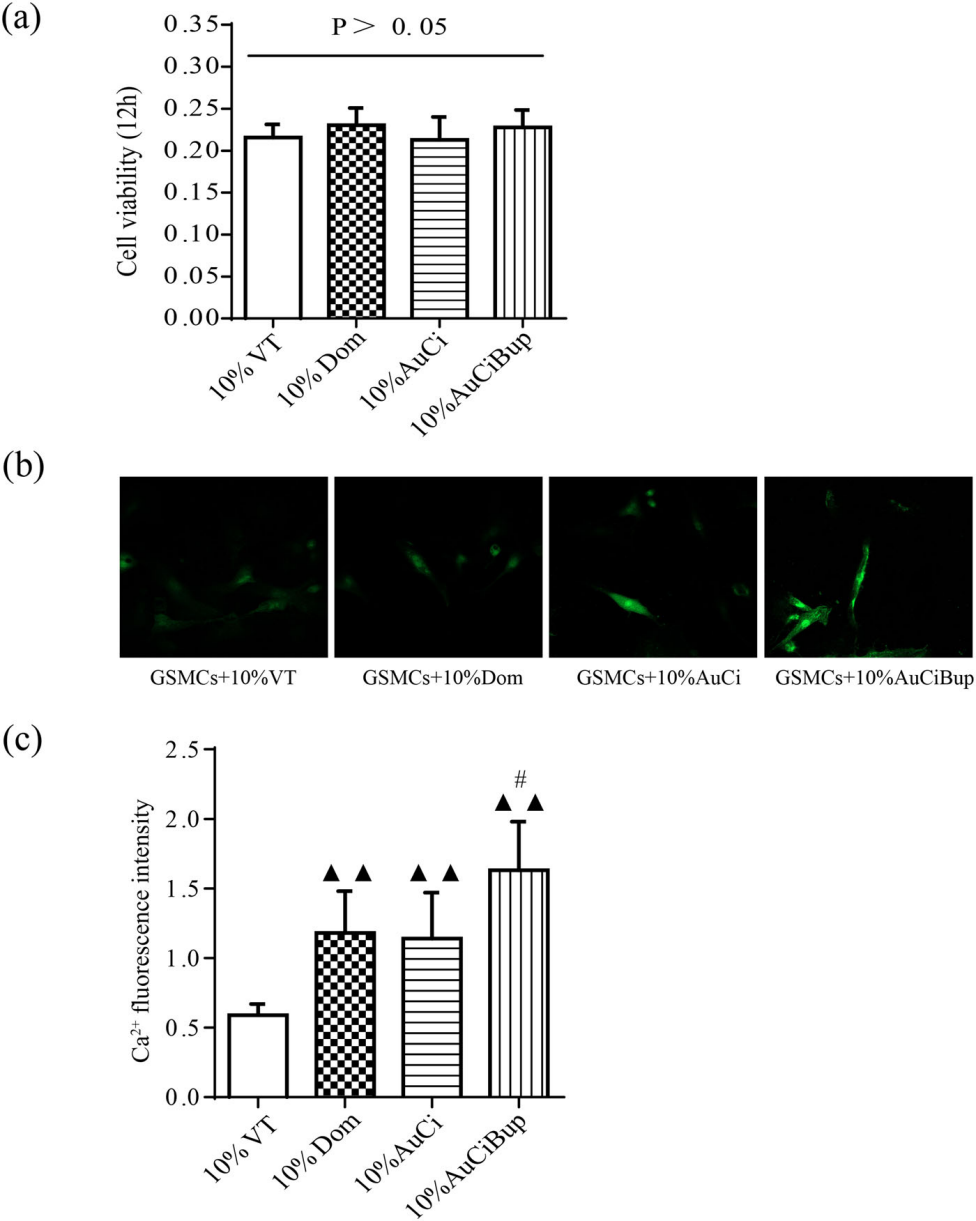
Effects of AuCi and AuCiBup on intracellular Ca^2+^ concentration in GSMCs. (a) The viability of GSMCs was assayed with a CCK-8 kit after treatment with 10% medicated serums of vehicle, domperidone, AuCi, AuCiBup respectively for 48 h. (b) The fluorescent image of free calcium ion in GSMCs after exposed to medicated serums for 150 s. (c) Increase of the intracellular Ca^2+^ fluorescence intensity after medicated serum treatment. The values are presented as the means ± SDs. ^▲▲^*p*< 0.01, compared with 10% vehicle treatment; ^#^*p*< 0.05, compared with 10% AuCi treatment. *N* = 5.

The GSMCs were successfully isolated and purified from rat stomach as observed under an inverted microscope (Figure 7(a)). The IFA with α-SMA as a specific marker depicted the characteristic of GSMCs and proved their authenticity in Figure 7(b). The viability of GSMCs after 5%, 10% and 20% medicated serums of AuCi and AuCiBup treatment for 12, 24, 36 or 48 h, respectively, was estimated with a CCK-8 kit to determine a medication dose and duration for the following intracellular Ca^2+^ assay. The unimpaired cell viability revealed 10% medicated serum treatment in 12 h was an optimal medication on GSMCs (Figure 8(a)). All 10% medicated serum treatment promoted calcium ion influx as displayed in Figure 8(b), whereas those of domperidone and AuCi enhanced 100% more fluorescence intensity and AuCiBup enhanced 200% than vehicle-treatment serum (*p*< 0.01, Figure 8(c)). Meanwhile, AuCiBup medicated serum significantly improved intracellular Ca^2+^ concentration in comparison with AuCi medicated serum (*p*< 0.05).

## Discussion

Accumulating evidence has proved FD is a non-organic disease. As the underlying causes of impaired gastrointestinal motility remain unaddressed, no cures are available at present and clinical care is generally limited to symptom management. Totally, prokinetic drugs are widely administered on FD patients to improve gastrointestinal disability. In contrast with mono-therapy, we pay particular attention on multidrug treatment with Chinese herbal medicines. *Citrus reticulata* and *Citrus aurantium* are herbal prokinetic drugs with curative effect on DGE. In Chinese classic formula of traditional medicine, *Citrus reticulata* and *Citrus aurantium* are generally prescribed with *Bupleurum chinense*, which is an antidepressant herbal drug. The underlying mechanism is still inexplicable in a modern scientific way.

5-HT is a biogenic amine with diverse effects in the central nervous system as well as in the periphery. It is mostly noted for its role in the pathophysiology of depression. Nevertheless, approximately 95% of the body’s 5-HT is synthesized and located within the gastrointestinal tract. The 5-HT receptors are expressed by neurons in the central and peripheral nervous systems and multiple subtypes have been distinguished. 5-HT_3_R and 5-HT_4_R are stimulatory subtypes in the enteric nervous system (ENS) and their activation enhances neurotransmitter release and propulsive motility patterns. Due to the low affinity to 5-HT of 5-HT_3_R (Taniyama et al. 2000), most studies highlight the prokinetic effects of 5-HT_4_R activation on the GI tract (Gwynne and Bornstein 2019; Galligan 2021). 5-HT_4_R agonists have literally been utilized clinically for almost three decades to relieve symptoms of constipation. 5-HT_4_R are G protein-coupled receptors that link to the stimulatory protein Gs which activates adenylate cyclase to increase intracellular cyclic AMP which then activates protein kinase A (PKA). Considering some antidepressants bring relief to millions of people suffering from gastrointestinal disorders, we propose herbal drugs specific to psychosomatic illness might activate 5-HT_4_R via promoting 5-HT release. *Bupleurum chinense* as an herbal antidepressant has been pointed out to modulate 5-HT function (Sun et al. 2017). So here, we address a combination therapy for functional gastrointestinal dysmobility by coordinating Bup with Au and Ci (herbal prokinetics) to formulate a multiherbal drug.

Interstitial cells of Cajal are derived from mesenchymal precursor cells that express c-kit, a receptor for stem cell ligand. Therefore, ICC can be identified with c-kit immunoreactivity. ICC associated with the myenteric plexus generate pacemaker activity in the form of slowly propagating waves of depolarization that provide rhythmicity and the direction of propagation to the slow-wave-driven motor patterns of the small intestine (Huizinga and Parsons 2019). Lack of normal pacemaker activity generated by ICC could account for symptoms of intestinal obstruction in the absence of mechanical obstruction. A considerable body of evidence has revealed that intracellular Ca^2+^ oscillations in ICC periodically activate plasmalemmal Ca^2+^-dependent ion channels and thereby generate pacemaker potentials (Streutker 2003). Our study showed both AuCiBup and AuCi increased ICC numbers and extracellular Ca^2+^ influx which might act as the underlying mechanism of their prokinetic activity. Anoctamin-1 (ANO1), the most studied member of anoctamin family of calcium-activated chloride channels, is a prominent conductance in ICC and these channels appear to be involved in pacemaker activity and in responses to enteric excitatory neurotransmitters (Sanders 2012). It is reported that melastatin-type transient receptor potential channel 7 (TRPM7) is required for intestinal pacemaking activity (Kim 2005). Whether the prokinetic effects of AuCiBup and AuCi are associated with ANO1 or TRPM7 is still in further research.

This study performed a comprehensive assessment on prokinetic effects of AuCi and AuCiBup. Either AuCi or AuCiBup treatment substantially expedited gastric emptying, helped to gain weight and rubbed up sucrose water preference and locomotive action as well, which indicated both herbal formulae not only modulated gastrointestinal physiological function but also tempered the relevant metal stress. Their double efficacy attributed to the enhancement of circulatory and central levels of 5-HT, MTL and SP. These neurotransmitters or brain–gut peptides activated vital genes related to gastrointestinal motility, such as 5-HT_4_R. As a result, all the upregulation and activation subsequently caused calcium ion influx to a great extent into gastric smooth cells. Furthermore, our findings suggested AuCiBup was superior to AuCi in terms of serum levels of SP and 5-HT, 5-HT_4_R expression, Ca^2+^ influx. The molecular synergy mechanisms of AuCiBup on gastrointestinal motility indicate the efficacy with the combination therapy on ameliorating gastrointestinal symptoms and eliminating 5-HT-triggered pathogeny is promising to be achieved in clinical patients. The other aspects related to the mechanism involved in the synergistic effects of *Bupleurum chinense* needed to be clarified in the future research and the clinical dose of the plant should be explicit in order to reduce the toxic effects reported in literature (Wang et al. 2014).
